# Managing the design and implementation of an agrochemical central sample preparation laboratory

**DOI:** 10.1155/S1463924693000057

**Published:** 1993

**Authors:** Alan N. Mannherz

**Affiliations:** American Cyanamid Agricultural Research Division P. O. Box 400 Princeton NJ 08543-0400 USA


Journal of Automatic Chemistry, Vol. 15, No. 1 (January–February 1993), pp. 19–22

### Managing the design and implementation of an agrochemical central sample preparation laboratory

**Alan N. Mannherz**
*American Cyanamid Agricultural Research Division, P.O. Box 400, Princeton, NJ 08543-0400, USA*

### Introduction

American Cyanamid's Agricultural Research Division (Princeton, New Jersey) performs Research and Development activities to discover and develop novel and safe biotechnology and agrochemicals to be used in the following general applications: insecticides, fungicides, herbicides, and animal health and nutrition. High capacity *in vivo* and *in vitro* assays are routinely used to screen organic chemicals and natural products for utility.

The CL File (Cyanamid's Chemical Library) co-ordinates the availability and flow of samples for testing in the high capacity primary assays. This group manages the acquisition, registration, storage, distribution, and retrieval of chemical samples and the chemical-related data associated with those samples (for example, structure, chemical name, purity and solubility). Samples for testing are obtained from internal synthesis efforts or from external academic and commerical sources. The chemical structures and related data are entered into online graphical and text-based database management systems. The samples themselves are placed in high-density storage systems for permanent storage. Prior to storing the samples, an automated distribution system is used to ensure that all compounds go to the biologists for primary screening.

### Historical

Perhaps the most important of the Chemical Library's responsibilities is the distribution of sample to the biologist for testing. A steady and reliable supply of compounds to feed the high-capacity screens is critical to the discovery of new products.

Historically, this distribution of samples was accomplished by the following system: groups of 40–50 bottles were placed in a cardboard box. A 5 in × 8 in card, listing the registration numbers and screen names, was also put into the box. The box was delivered to the first screen and the appropriate biologist would specifically weigh the amount of sample that was needed, place the subsample into a separate container, write a label for this container, check-off the 5 in × 8 in card for that screen/registration number, and then, when all the samples had been weighed, deliver the box to the next screening group where an analogous procedure of subsampling occurred. When all of the screens had processed the box of samples, it was returned to the Chemical Library. The bottles were placed in storage and the checked-off card was placed in a notebook for future reference.

This system functioned admirably for almost 30 years. The circulation of samples required that a technician or scientist in each screening group spent approximately 40 man-hours per month on subsampling. It took approximately two to three weeks to completely circulate each box through all of the screening groups.

With the development of receptor and enzyme-based assays, the definition of high-capacity screening changed from approximately 10 000 compounds/year to 25 000 or more. Both plant and animal-related *in vitro* assays were being developed. A suggestion was made to management to co-ordinate the screening efforts of these two groups, perhaps automate the assays, and institute a centralized weighing facility to provide the increased number of samples that would be required. The sample preparation was also envisioned as an automated process. The logical location and management of a sample weighing/preparation facility was where the chemical samples themselves were currently located, in the Chemical Library. This suggestion was made on 1 May 1987. Thirty-four months later the first robotically-prepared solutions of organic compounds were delivered to all screening groups from the Chemical Library's Central Sample Preparation Laboratory.

### Preparation

The switch from a totally manual sample preparation system, uniquely performed by each screening group, to a centralized and automated system was not an overnight task. A change of this magnitude required considerable planning and discussion to assess the needs, logistics, and feasibility of such an operation.

It seemed logical that an automated system should mimic the manual system as closely as possible. However, initial concepts of having a robot transfer the chemical samples directly from the original storage bottles seemed very unlikely. The chemical samples were not necessarily homogeneous and varied in state from nice flowable powders/crystals to viscous liquids, gums, or pastes. Other features of the manual system (i.e. weighing,

This paper was read at the International Symposium on Laboratory Automation and Robotics (October 1992, Boston, USA).

© Zymark Corporation 1993

solubilizing and subsampling) seemed conducive to automation.

Once the concept was envisioned and the organizational responsibility accepted by the Chemical Library, a formal recommendation was made (October 1987) to form an investigative committee. The committee consisted of biologists from each screening group and representatives of the Chemical Library. If sample preparation was to be centralized and use robotics, then all recipients of the samples would have to agree to conform to a more standardized system of sample prepartion.

### Gathering data

The key issues which had to be addressed at the first committee meeting (December 1987) included the following: Who are the current internal and external users of robotics and automation? Can all aspects of the manual system (other than weighing) really be duplicated by a robotic system? Will all screens participate (i.e. *in vivo* and *in vitro*)? What automation hardware and software is necessary and appropriate? Will all samples (old and new) be tested by all screens? What solvent(s), sample size(s), and container(s) should be used? How should insoluble compounds be handled? How do you prevent contamination between samples that are processed robotically? What laboratory facilities are required? How should this operation be staffed?

In order to address these questions the Chemical Library devised a questionaire for each screen to complete (see figure 1). It was distributed at the second committee meeting in February 1988. The results of the questionnaire varied between screens. Weekly schedules of testing, numbers/types of samples tested, weights required, solvents and concentrations used, and type of vessel for sample preparation were all different.

*Figure 1. Central weighing screen questionnaire.*

Date:
Section:
Screen name:
Screen type (circle one): *in vivo in vitro*
Target organism:
Person in charge:
Total compounds per week:
Test days (circle *all* that apply) M T W TH F
Number of compounds tested *each* time:
Source of compounds:
How many samples weighed by your staff each week?
Test environment (plates, leaf dip, spray, feed, other?):
Sample preparation (wet, dry):
Weight of compound:
Solvent(s)/ratio:
Incomparable solvent(s):
Concentration/volume:
Type of vessel/size (250 ml flask, plate, other?):
*Do you currently piggyback off another screen?
If yes, which one?
*Do you plan to increase capacity or add new screens?
If yes, please specify.

Initially, the committee focused on developing a short-term plan for a central weighing operation (without robotics), believing that his approach would be easier to implement and the benefits would be quickly realized. However, the committee had some difficulty agreeing on staffing (finding someone who was willing to precisely weigh thousands of samples for all screens) and the type of solvent to be used (for example acetone, water or EtOH). If was not desirable to burden current (or new) staff with precise weighings for all screens and the committee could not agree what the most common solvent (or combination thereof) that could be used. However, after the third committee meeting (March 1988), it was decided that one full-time person (under supervision) would be required to precisely weigh/ prepare samples, dry samples would be given to the *in vivo* screens, and DMSO would be used to prepare samples for the *in vitro* screens.

The DMSO-prepared samples would be weighed into (Radio immuno assay) (RIA) tubes and solubilized by a Cetus Propette. In the future the RIA tubes would be handled by a modified Zymark robot which used Hewlett-Packard software. This robot had been recently purchased by one of the biology groups to run a receptor screen.

A formal proposal was made (May 1988) to management for the acceptance and establishment of a central weighing facility by third quarter of that year. In August of the same year, a new-position job description was submitted for approval. However, the momentum for this project seemed to wane. As of November 1988, no affirmative reply was received to begin implementing the new system. Two major concerns remained unresolved. In the interim, several biologists and the Chemical Library continued to address these issues: pursue the goal of a common solvent and fine tune the vision of a centralized operation (without precise weighings).

One possible source of information was from those individuals that were currently using robotics. The ideal was to find someone with a similar application. Within the Agricultural Research Division there were two robots: one Zymate I Robotic System (purchased in 1985) was used to extract medicated feed and the other, modified, Zymark system (purchased in 1987) was used for receptor assays. Contacts were also made with other companies, such as duPont, Monsanto, FMC, and Eli Lilly. At that time, only Eli Lilly had a similar operation and a visit was made to Eli Lilly's Greenfield Laboratories in Indiana in December 1988. Eli Lilly used a robot in one of their biology groups for making solutions to a known concentration. Sonification was used for dissolving solids and the robots removed aliquots from the stock solution for various assays. If a screen wanted a different solvent (other than 90 parts acetone/10 parts EtOH), the sample was dried and the appropriate solvent added. Precisely preweighed, dry samples were supplied to the screeners by a centralized weighing operation.

Prior to this visit, two major decisions were made that finally resolved the last two concerns of management for a centralized, sample preparation operation:

(1) It was not acceptable to precisely weigh every sample manually. The most desirable concept, then, was to design a system that was based on volumetric rather than gravimetric principles. This would allow samples to be prepared in solution without precise weighings and seemed amenable to the use of robotics.
(2) The problem of finding a common solvent was solved. Since each screen used a different concentration and solvent (or solvent mixture), samples robotically prepared had to be in a solvent that readily evaporated as well as dissolved most organic compounds; 100% acetone was decided upon.

The most important factor in sample preparation was to provide the screeners with the appropriate amount of technical material, whether, it was in solution or in suspension. If the solvent evaporated, the technical material would still remain. In fact, this was advantageous to those *in vitro* assays that required DMSO. A 'dried off' microtiter plate/vial could be easily resolubilized. The volatility of acetone was also helpful since the technical material did not remain in a solvent very long and therefore there was less concern for long-term stability if the samples/solutions had to be stored.

During this time (1987 to Fall 1988), information was being collected on the current vendors of robotic equipment and systems. A literature search was performed. Companies such as Fischer, Perkin-Elmer, Hewlett-Packard, Source For Automation, and Zymark were identified and contacted. Company literature was obtained and reviewed. Each vendor offered a unique robot with some add-on modules to perform different tasks. At that time, the vendor who offered the greatest flexiblity in automated systems and who provide us immediate technical expertise was Zymark. Two meetings were held with Zymark's Technical Representative in September and October 1988; after the second meeting, Zymark produced a detailed breakdown of what was needed and how much it would cost.

It was then possible to hold a meeting of screeners and management (in January 1989), a detailed presentation was given of how the robotic system would operate, how data and samples would be handled, and (most importantly) what commitments would be needed for the screeners to truly standardize such an operation. Those commitments included:

(*a*) Samples to be delivered as solutions in one solvent system to all screens on a regular schedule.
(*b*) Backlog of solutions to be maintained for some period prior to actual testing.
(*c*) Same compound in solution prepared for all screens, regardless of chemical structure.

As a result of that meeting, a human-robotic exercise was undertaken for three weeks. One of the biologists, acting as much like a robot as possible, prepared acetone solutions of chemical samples for all of the screens. This exercise helped to determine if there were any unforseen problems. Some of the conclusions were that central weighing must involve a human making an approximate weighing; robotics could be employed in making solutions and dilutions, albeit sonication and vortexing were not adequate to create suspensions of insoluble compounds; and the delivery of solutions each week, automatically, to the screens would increase the efficiencies of those screens.

On 10 March 1989, the Chemical Library was given the go ahead to implement the new system.

### Implementation

One of the first considerations was: where should the operation be located and what laboratory facilities would be required? The first request to the Facilities Manager for laboratory space was made one week *prior* to the announcement of the committee's formation (October 1987). A new R&D wing was scheduled to be occupied in 1989. The Chemical Library was moving to the new wing and did not have sufficient space allocated for a centralized sample preparation operation. The operation was not envisioned many years earlier when space was being allocated in the new wing. The older R&D facilities that were going to be vacated were also scheduled to be renovated. Therefore, there was a scramble amongst the researchers to claim (and justify) space in the renovated laboratories.

Throughout the planning process, discussions were held and written requests forwarded to the various facilities managers. Temporary laboratory space and use of equipment were constantly being negotiated with the biologists. Fortunately, a renovated laboratory (about 22 × 30 feet), close to the new location of the Chemical Library, was approved in September 1989. This provided sufficient space for the first (and second) robot, as well as the opportunity to design a laboratory especially suited to the new operation. The newly renovated lab was occupied early in Januray 1991.

The request for addition to staff was also approved in 1989. A job was advertized in June for a person with an A. A. S. degree or with two years of college education in a physical or biological science. A suitable candidate was identified and joined the group in October, one month prior to the delivery of the robot. Immediate supervision (project leader) of the new operation was given to an existing member (a B. S. Chemist) of the Chemical Library who had some computer and database management experience. The Project Leader attended Zymark's basic Pytechnology training course (September 1989); the robotic technician attended the same course in January 1990.

According to Zymark, the application was relatively simple to adapt to robotics. Working with the technical representative and their system engineers, a viable scenario of events was conceived:

(1) Robot places empty, capped test-tubes (from rack) onto balance, computer calculates tare weight.

(2) Robot places empty, capped tubes in rack.

(3) Technician adds estimated amount of sample to tubes.

(4) Robot reweighs tubes, computer calculates net weight of samples.

(5) Computer calculates amount of solvent necessary to make a constant concentration for each sample.

(6) Robot uncaps first tube.

(7) Robot adds correct amount of solvent.

(8) Robot carries tube (solution/suspension) to homogenizer and homogenizer stirs (and grinds) the sample.

(9) Robot returns tube to rack and homogenizer generator is cleaned.

(10) Robot subsamples solution/suspension into three to four different uncapped vials/microplates.

(11) Robot caps tubes/vials.

(12) Robot picks up next tube and repeats procedure starting with step 6.

Each robotic operation required a certain amount of time to execute. Since it was decided to process 40 samples per robot run, these individual times were increased 40-fold. The estimated time for one run was about 11 h. So the utility of robotics versus a manual system was questioned: a person could probably weigh tubes, add solvent, etc. more quickly than the robot. It was necessary to supply the screeners with samples in a timely fashion and so would need the capability to do more than one run per day. In order to save time several changes were made, including: an inverted slip cap on the screen vials was used to reduce evaporation of acetone instead of a threaded cap; the Pysectors were rearranged for the most time-efficient orientation between modules; and Zymark's System Productivity Software was purchased to enable the homogenizer generator to be cleaned while the robotic arm continued to subsample solutions.

Processing insoluble samples posed a unique problem. The use of a solvent for all screens was limited to acetone. The addition of small percentages of DMSO, $H_2O$, EtOH, or MeOH could not be tolerated by some of the sceening targets and/or methodologies. The initial experiments (human-robotic) indicated that sonification and vortexing were inadequate. These methods did not break-up or disperse insoluble compounds in the acetone. One of the screening groups regularly used a Brinkman Polytron homogenizer to grind up insoluble compounds in solution for use in a spray chamber—if a true solution could not be sprayed, then at least a microfine suspension would be sprayed. This approach seemed like a possible solution for the preparation of samples. Zymark was able to incorporate a homogenizer into the system and provide it with necessary self-cleaning capabilities.

We looked at Zymark's Zymate II PyTechnology System Configuration with them—some Pysectors were standard modules, while others required customization. Discussions were held with the system engineers and some of the modules were demonstrated. Every item on the equipment list came under close scrutiny and generated many questions; it was a definite advantage to see the modules in action.

Many more final decisions had to be made during the following weeks such as: what size/type of test-tube should be used and what size/type of vial should be used? By the end of May, an official system quotation was received from Zymark. The Zymate II system was ordered in July, and it was received on 1 November 1989.

During the next couple of days the system was assembled. During the next four months, the system was debugged and validated. The implementation of the new sysem did not occur as smoothly as had been anticipated. Some of the problems encountered include: original estimated time per run was 6·33 h (actual time was 10–11 h); the 10 ml pipette tip occasionally missed the confirm button, or, when it did hit the button, no signal was transmitted; pipette tips would drip; and the pipette would not go to the bottom of the test tube and take up the correct volume of solution every time. Working closely with the system engineer and field service engineer, many of the problems were solved. On 24 February 1990, the first group of robotically prepared solutions were delivered to the screeners.

### Conclusion

The advantage of centralizing an automating the sample preparation system are numerous In addition to expanding the responsibilities of the Chemical Library, some of the advantages are as follows:

(1) Increased screen productivity since the biologists now have additional time to carry out their professional assignments.

(2) Reduced sample loss and chance of contamination, since only one weighing is made for primary screens rather than several separate weighings.

(3) Reduced number of manual weighing errors since the robot samples volumetrically.

(4) Minimization of lost or misplaced sample bottles—since compounds remain in the Chemical Library area rather than being circulated.

(5) Faster sample turnaround because the handling of samples is limited to Chemical Library personnel.

### Acknowledgements

An effort of this magnitude requires, the time, sacrifice, and co-ordination of many individuals. Everyone at the Agricultural Research Center who contributed towards the development and implementation of this operation are to be commended. Without the support of key Zymark personnel, the envisioned system would not have materialized.

